# Supplementary material for: Acceptance and compliance with micronutrient powder (MNP) among children aged 6–23 months in northern Nigeria
Source: PLOS Glob Public Health. 2022 Oct 17;2(10):e0000961. doi: 10.1371/journal.pgph.0000961 (PMC10022258; doi:10.1371/journal.pgph.0000961)
Supplement: S1 Dataset — (ZIP) [file pgph.0000961.s008.zip › S8_Dataset/In-depth Interviews.zip!/In-depth Interviews/080715_K_J_AD_2_CG.pdf]

INDEPTH INTERVIEW WITH FEMALE CAREGIVER USING MNP IN JEGA LOCAL GOVERNMENT AREA OF KEBBI STATE

INTRODUCTION

I. Good morning ma, could you please describe for me the people living in your immediate household?

R. In this house we are very plenty, I do not know how I am going to start counting us for us because this is a large house. You see on this our side we are much on the other side (points at another building) they are also there so I cannot start counting how many people are in the house.

I.Ok thank you but can you tell me in this particular house i.e the house you are with your immediate family how many people reside here both adult and children?

R. There is our husband, I, my co wife, I have 4 children and she also has four children.

I. Can you please tell me how many of these children are between the ages of 6 to 23 months?

R. it is just this one in my hand, my co wife all of her children are grown up in fact some are even married. He is just a year old.

CHILDREN 6- 23 MONTHS

I. Thank you very much, previously when we were here we heard about some food challenges related to seasons, could you please tell me about how you cope with them?

R. I do not have any problem with that because what I usually give to my baby is tuwo and kunu and there is always kunu in the house so it is not a problem.

I. One thing I heard in other interview is that caregivers give their children "tea", I would like to know what tea means please?

R. Habamallama don't you know what tea means, you know now after all is it not you people that even drink tea all the time (laughing)

I. Yes you could be right but you know there might be different ways of making tea, I just want to know how you make your own tea in this community.

R. Ok, usually we put milk, bournvita (cocoa) and sugar in hot water. That is how you prepare tea and take it with bread if you have if not you can just drink it like that.

EXPERIENCE WITH MNP DURING TRIAL

I.Thank you very much, now I just want you to tell about your experience with the MNP that was given to you. Tell me your story about it.

R. The medicine is good, I am very happy with it. You know I just started using it not long ago but sincerely I am beginning to see some changes in my baby, he eats more food now and he has grown fatter, can't you see him. His appetite has increased a lot, I am very happy gaskiya.

I. that's good mallama but apart from his appetite improving is there any other thing you enjoyed while using the MNP?

R. Tohmallama I have told you all now, I did not have any problem with the MNP, it was easy to use and my baby did not reject it he is taking it and now he likes to eat and eat and eat. I will continue giving it to him by God's grace.

CHALLENGES/BARRIERS USING MNP

I. Please I would like for you to tell me about any barrier you faced while using the product?

R. I did not face any challenge o, like I told you earlier it was easy to use I did not face anything problem at all, what challenge in this small thing nooooo, no problem at all, I did not face any problem.

I. You know there were some caregivers that complained that it was difficult for them to store the product with rodents eating it or others coming to take it. I would like to know your experience on such?

R. I did not experience anything like that so, did you people not instruct us to keep it where children cannot get to or where rats can also not get too. I kept it on top of my wardrobe, I have rats in my house o but even for once they have never gotten close to the medicine.

I. I sincerely appreciate the way you have been discussing with me my sister but now I would like for us to talk about any side effect that you think was caused by the product?

R. Did anybody tell you about any problem that her child had after taking the drug?

I. No I did not say so, I just want to know whether there was any problem you noticed from your child?

R. I did not notice any problem with my child, like I told you earlier on the baby eats more now and has put on more weight so and I believe that is a good thing. He has not rejected the drug and since he started taking it he has not even been sick even for one day.

I. There were some mothers that said sometimes it could be difficult remembering to use the MNP, did you have any similar experience?

R. I did not experience something like that, how can you forget to give your child what is meant to help him. You know some women can be careless so they would not pay as much attention as they ought to. For me I did not forget even for once, the first food I give to him will have the medicine inside of it.

I. what advise can you give to these women that experience such challenges?

R. I don’t have any advice for them, since they choose to be careless about the wellbeing of their baby am I the one to care for them, how would you be given something that will help your child free of charge and you would not take it serious?. I do not have any advice for anybody.

I. Thank you very much but please lets assume that I am facing some of this challenges, what would you advise me to do?

R. I would advise you to try and keep the product where you know you can see it all the time but also it should be where other children cannot get to it, like me I told you I usually keep it inside a plate on top of my word robe, you see none of my children can get there and rats to cannot get there. The word robe is also facing my bed so once I wake up in the morning I see it. That way I would remember to give the child the medicine to take.

CHILD FEEDING BEHAVIOUR AND RESPONSE TO MNP

I. Previously you mentioned that you child mix the product with food and give to the baby, could you please tell me what food you usually mix the product with?

R.I mix the medicine with kunu and give to the child to drink, usually I will fetch some of the kunu inside “ludayi” (local spoon used in drinking kunu and fura), mix it with the medicine and give him to drink once we are done then I give him the remaining kunu in the bowl to drink.

I. The kunu you give to the baby is it cold or hot?

R. I mix it with kunu that is not very hot.

I. What time of the day do you usually give the MNP to the child?

R. I told you that is the first food I give to him in the morning.

I. Ok thank you very much mallama. From your own experience do you think the baby notices that the kunu was mixed with something else whenever he takes it?

R. No he doesn’t notice anything in fact once I just put the “ludayi” inside his mouth he would just drink it direct, he doesn’t notice anything at all.

I. Previously you mentioned that his appetite has increased and he eats more now, could you please tell me what kind of food he eats more of now?

R. He eats more of tuwo andkunu now.

I. Is there any other food that he eats more of now?

R. Those are the food we have so that is what he eats.

I. What about breast milk?

R. He still takes breast milk as usual, right from time he drinks a lot of breast milk so I cannot say really.

SHARING MNP

I. In other communities, the sharing of MNP was noticed, I would like for you to please tell me your experience about that?

R. I did not share the medicine with anyone, I remember I was told that I should only give it to the boy and that was exactly what I did, even when the other kids wanted to taste it I told them no it was not meant for them. Nobody apart from the boy took it.

I. if I should get you correctly even you did not taste the MNP

R. Well the first day I used it I just taste some so that I would know what I was giving my child you know but apart from that day nobody else has tasted it apart from the baby.

I. Thank you very much, now if the child doesn't finish the kunu that is mixed with the MNP what do you do with the rest?

R. Usually he finishes it because like I told you I first mix it with small kunu in the "ludayi" before I now give him the rest of the kunu to drink, so he usually finishes it.

I. You were given 60 sachets and told to use one per day right, well I want to know in your own opinion whether you think these 60 sachets would be enough for 60 days?

R. Of course why not, am sure you people that made it know why you put it that way, I cannot say it is not enough because I did not go to school so I do not know what is inside but since you people say we should give them one per day then I think it is ok.

PERCEPTION OF MNP

I. I noticed you kept calling the product a medicine, I would like for you to please clarify for me whether you see it as a medicine or as food?

R. I see it as a medicine now, because you said we should be mixing it with food and give to the child, how can you mix food with food again, it is medicine you mix with food.

WILLINGNESS TO PAY FOR MNP

I. Am sure you were told this product is for free but what if you were asked to buy it, do you think you can buy it for some token amount of money?

R. Yes why not, it is helping my child a lot so if the father would agree to buy it for him why not, I would tell him to buy it.

I. So how much do you think you can buy the product?

R. I cannot say because I do not know how much you people would say you want to sell it, if I can afford it then I would buy if not I would leave it.

I. Well yes I understand what you are saying but I just want to know how much in your own opinion now you think the product should be sold?

R. It should not be more than N200.

SOCIAL MARKETING THE MNP

I. Thank you very much my sister for your time. Could you please tell me some ways you think would be effective for promoting the MNP in this community so that they would comply with using it appropriately?

R. When you bring the medicine you would explain to people how to use it just like the way you explained it to me.

I. What people must influence your opinions and knowledge about feeding practices?

R. Our mothers are the ones that teach us how to care for our children especially if you are a new mother, your mother would come and help you and teach you how to take care of the child.

I. Ok, thank you very much but I would like to also know who influenced your decision to give the MNP to your child?

R. It is my husband, if he doesn't give permission I have no right to do anything, he has to approve it first.

I. Where would be a good place to disseminate health information related to MNP?

R. It should be through the village head because we trust him so if he says something is good then we would accept it because we know he cannot cheat us. Once you bring it to him and he announces we would come and collect it.

I. Who in your community do you trust the most for receiving information about infant and young child feeding?

R. It is like you are repeating this question because I have answered you before, I told you that it is from our mothers they are the ones that usually put us through on how to feed your baby or sometimes if your mate (other wife) wants to help you she would also help you.

I. Ok thank you very much my sister but what I am just trying to find out is if for instance now you are looking for more information about how to feed your baby with nutritious food that would help in building his body where would you go to?

R. We go to the health center, that is where sometimes we get educated on the kind of food to feed our children that would not make them have tamowa (malnutrition) and if the child already has it then they would give you "madarantamowa" (RUTF) to give to the child.

I. What forms of media do you prefer for receiving MNP messages?

R. Through the village head and then through the health workers at the hospital.

I. What about through brochure/flyers/posters?

R. Yes poster is ok with pictures so that we can see it, like the poster they usually show us of how to take care of your baby during antenatal. yes poster is good.

I. how about through word of mouth i:e from one person to another?

R. Yes it is also ok to, like if somebody should ask me now I can tell her about this medicine because I have used it and it is good.

I. Do you think passing messages through the radio would also be effective?

R. Well it is good but how many people have radio, it is only those that can afford radio that have radio but you see if you bring it to the village head he would send the town announcer to go round informing people and everybody would be aware.

I. ok thank you very much for that information. Please I would like to know about any other health education campaign that was carried out in this community?

R. I do not know.

I. I just want you to tell me about any health education campaign in your community that has taken place and how it was?

R. Please I do not know mallama.

I. Ok thank you very much, we are almost through please. What are some of the ways you feel are the channels to distribute the product?

R. I have told you already, through the village head, once you come to him that is all or was it not through him that you people came the first time.

I. That's true, thank you my sister, could you please tell me some specific messages that you think would be effective in promoting the product?

R. You would explain to the person the importance of the medicine to her child and that she should give it to the child without sharing it with other children or else it would not work for that child it was meant for?

I. Oh that's so good, ok lets say you want to explain the product to me and how to use it, what would you tell me?

R. (laughing) I would tell you to give one sachet per day to your child, you should mix it inside kunu that is a bit warm and give to the child. Make sure that you mix it with the kunu you are sure the child can finish because if he doesn't take all it would become a waste. Then I would tell you to keep it in a place where the other children or any other thing like rat or cat would not be able to touch it. I will say do not share the medicine with anybody even if the other children

should cry you should explain to them that the medicine is not meant for them but for their brother/sister so that he would also grow like them.

I. Ahh my sister you have really tried, I sincerely appreciate you a lot, now we have come to the end of this discussion but before I gois there anything you would like to share with me about your experience since you started using this product?

R. I do not have anything more to say because I believe I have told you everything, the medicine is good because it has made my baby bigger and he eats more now so I thank you people for giving it to me may God help us all thank you.

I. Amen and thank you for thanking us too, make God help us all. One more thing before we go please, what advise can you give us on how to make this program more effective?

R. I do not have any advice mallama, all you people have done is good thank you.

I. Thank you and do enjoy the rest of your day.
